# Supplementary figures and images for: Puncturing apple fruits increases survival of Grapholita molesta (Lepidoptera: Tortricidae) in laboratory rearing
Source: PLoS One. 2022 Apr 29;17(4):e0267890. doi: 10.1371/journal.pone.0267890 (PMC9053792; doi:10.1371/journal.pone.0267890)

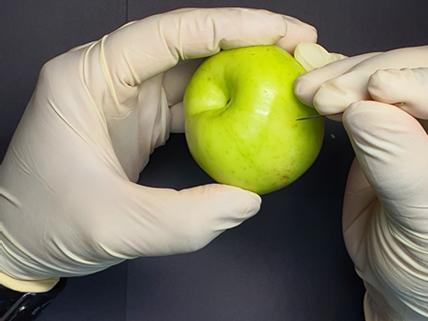

Supplement: S1 Appendix — (TIF) [file pone.0267890.s002.tif]
