# Supplementary material for: Puncturing apple fruits increases survival of Grapholita molesta (Lepidoptera: Tortricidae) in laboratory rearing
Source: PLoS One. 2022 Apr 29;17(4):e0267890. doi: 10.1371/journal.pone.0267890 (PMC9053792; doi:10.1371/journal.pone.0267890)
Supplement: Attachment — Submitted filename: Gm Response to reviewer 13Apr.docx [file pone.0267890.s003.docx]

**A. Response to reviewer #1’s comments**

1. This study showed the needle punctured green (?) apple could be a better substrate for Oriental fruit moth larval rearing medium than not-punctured ones.

The experiments were well designed and conducted. Writing is concise and without redundancy. Results were reasonable. It is difficult to judge the scientific merit of this finding in science but surely important in applied aspects.

>> Thanks for the comments.

2. M&M Please provide when and how did the field collection of apple was done and detial the condition of apple collected.

>> We provided the time and collection procedure in the revised MS. Please see L80-82 in the revised MS.

3. Table 1. egg duration was presented. However in M&M section, larvae were introduced into the experimental apples and began observation. This part could be cleared.

>> We addressed this issue in our previous MS. See L71-75 in the revised MS.

4. In Table 1, provide the actual numbers of observation for each stage.

>> Yes. Please see Table 1 in revised MS.

5. Exiting rate was measured but not described in M&M. Provide the details of the measurement.

>> We corrected the sentence by adding “exiting larvae”. Please L95-96 in the revised MS.

6. In the box, if the larvae exit the fruit then, does this mean that the larvae became dead, on what time scale?

>> We addressed this issue in our previous MS in L93-94. When a larva exits from the fruit, it transferred into another dish for pupation. Please see L95-98 in the revised MS.

7. please check the statistics; the rate larvae exited from fruits (Zc = 1.83, P = 0.067), the pupation rate (Zc = 0.99, P = 134 0.324), and the adult emergence rate (Zc = 0.00, P = 0.000) were also not different between punctured and unpunctured apples, Authors discussed the punctured apple could provide easy approaches to the week larvae to enter into the fruits and lowering larval mortality.

>> As we mentioned in our previous MS in L184-185 and L202-204, the overall survival rate and entering rate of the 1st instar was higher in punctured apples despite the lack of significance in each parameter, i.e., exit rate of larvae, pupation rate, adult emergence rate. Please see L185-186 and L203-204 in the revised MS.

Nevertheless, we deleted Figure 2 as it is redundant data of Table 2.

8. In the study, separation of larval mortality and exiting rate, and pupal mortailty and pupation rate was not explicitly detailed, and which made the overall conclusion less supporting.

>> Please see our answer to above comment.

9. In the table showing adult reproduction, please provide the numbers of female observed for oviposition.

>> Yes. Please see L110-111 in the revised MS.

10. In this study, how many pairs (male and female) were studied were not presented. If If there was uneven number of male and female produced from the treatment, did you add the male from the rearing batch or ...

>> We addressed this issue in L110-113 of the revised MS.

11. Authors had narrowed the scope of this finding only in laboratory rearing, however this findig could influence field dynamics of infestation.

>> We agree with the reviewer’s comment. We added “However, in field condition, physical damage on apple can negatively influence the population dynamics of *G*. *molesta* by plant volatiles produced to attract natural enemies” in the revised MS. Please see L205-207 in the revised MS.

12. By puncturing, the host apple could provide more VOCs which would influence the host location to the larvae. Please add the possibility of this in the discussion, relative to the field situation where diverse factors could impact apple fruits and give physical damages.

>> Please see our answer to above comment

13. In this study, can we have any data of apple physical character change over the experimental periods? Since the larvae should stay ap 2 weeks inside the host food once it entered into, physical changes of the host food could influence the outcome of the results.

>> We addressed this issue in our previous MS in L206-208. Please see L209-211 in the revised MS.

**B. Response to reviewer #2’s comments**

1. In this work the authors study the effect of punctured apple fruits on survivorship of G.molesta. Based on their analysis the authors conclude that punctured fruits are more suitable for OFM laboratory rearing compared to non-punctured fruits and as it is shown by the demographic parameters that the authors have estimated including the intrinsic rate of increase.

These results support previous works done in G. molesta and are generally interesting and important for OFM rearing. In this respect the authors reaffirm the importance of their work to the fact that their results will contribute to the mass production of this pest to be used for mass rearing. On the other hand, I feel that the current work is rather simplistic and based on a rather small data set.

1. I cannot imagine also how practical might be the mass production of OFM in a natural host compared to artificial diet despite the better demographic performances of the species. For example, injured apples will not only improve OFM growth (at least as the present work demonstrates) but also the growth of fungal infections and ultimately cannot be used for a long time being impractical. i.e. How many times have you changed the apples during larval development? How easy it is to mechanize the technique you are proposing for OFM mass rearing?

>> We agree with the reviewer’s concern that this rearing method using natural host can’t be appropriate for commercial mass-production. This is why we just suggested that punctured apple is better than unpunctured apple throughout MS although Vetter et al. [25] found that the punctured-apple diet produced more pupae than the artificial diet.

Nevertheless, we added a sentence in result section regarding fungal contamination on punctured apple. See L143-144 in the revised MS.

2. Additionally, the study does not examine whether the ''good reproductive performance'' that the authors have observed (through the two comparisons: punctured vs unpunctured hosts) holds also for other temperature regimes. Because the intrinsic rate of increase takes in to account at least both, the net maternity along with survivorship of the individuals of a cohort, it is quite possible that the differences might vanish under different temperature regimes. In table 4, for instance, the distance in rm between the two treatments is considerable shorter compared to Ro. Actually, I consider the intrinsic rate of increase as more representative for the description of the reproductive success of species (i.e. fitness) compared to the net maternity and not exclude that this difference might diminished after a slight alteration of the temperature regime.

>> We agree with the comments of the reviewer. Different temperature regimes may have an impact on *G*. *molesta*'s development as well as its intrinsic rate of increase. However, our goal was to see whether puncturing apple fruit might improve the intrinsic rate of increase or not. That is why we maintained the same environmental and fruit conditions.

3. Finally, the authors focus on the technical part of their study for OFM rearing (which is sound and not bad), but, to my view incomplete because they have not examined in deep why they have observed such significant differences. Particularly and if I am not wrong, a part of the last lines (213-214), there is actually no hypothesis of what is the profound reason-interpretation of their result and especially what is(are) the main reason(s) that the species explicit different fitness capacities when feeded with an injured host.

>> We changed “Therefore, in this study our goal was to compare the effects of punctured versus non-puncture apple fruit on the development, reproduction, and life table parameters of *G*. *molesta*.” to “Therefore, in this study, we hypothesized that punctured apple fruit may increase fitness of *G*. *molesta* by enhancing development and reproduction. We compared biological and life table parameters of *G*. *molesta* reared on between punctured and non-punctured apple fruits.” Please see L54-57 in the revised MS.

4. If it’s just the fact that it’s easy (and not energy consuming) for the larvae to crawl insight an injured apple compared to an unpunctred one, how do they explain the differences that are observed on the successive developmental stages? Are they excluding any nutritional, physiological or any other cause? Thus, I believe that the biological-ethological and consequently the ecological dimension of the authors' findings is of greater interest but not emphasized at all.

>> We thought punctured apple should be artefact thus it may not easily applicable to natural interpretation. Anyway, we added a discussion on impact on field population as suggested by reviewer 1. See L205-207 in the revised MS.

5. Line 45. If this proposition were to the maximum, i.e. biological was effective, we would not need research on pesticides or even deprivation.

>> We corrected the sentence by adding “potentially” in the revised MS. Please see L45 in the revised MS.

6. Line 58. It would help the reader if you included a photo in the appendix

>> We added a photo in the appendix demonstrating the puncture to the apple fruit. Please see L372-374 in the revised MS.

7. Figure 1. To be honest I cannot follow your chart. Why do you not just plot the survivorship curves for each stage and treatment?

>> Yes, we revised the figure and caption. Please see Figure 1 and L363-364 in the revised MS.

**C. Response to reviewer #3’s comments**

General comments

1. This is a study where unpunctured and punctured apples were compared as diet for artificially rearing the pest Oriental fruit moth, Grapholita molesta. To determine the effect of these two diets, authors measured and analysed some demographic parameters in the reared insects. The article has some interesting results. However, the results are not novel and, results are poorly presented. The manuscript has a very narrow scope, basically in the field of entomologists. My major concern is the experimental design since there is not explanation of what the experimental unit was, the apple or the insect.

>> Apple fruits were the experimental unit and we revised the sentence. See L81 in the revised MS.

Introduction

2. Lines 46-47. A mass-rearing system based on apples. It is not possible. Fruit phenology and fruit variation surely will introduce many difficulties in the process.

>> We agree with the reviewer’s concern that this rearing method using natural host can’t be appropriate for commercial mass-production. This is why we just suggested that punctured apple is better than unpunctured apple throughout MS although Vetter et al. [25] found that the punctured-apple diet produced more pupae than the artificial diet. Anyway, we deleted “mass” from the revised MS. Please see L48 in the revised MS.

Methods

3. Line 67. How many insect per cylinder?

>> We deleted “they” and added “10-15 pairs of adult moths” in the revised MS. Please see L68 in the revised MS.

4. Describe with detail the rearing method. Did the authors place the larva on apple surface? How did they do it?

>> We corrected a sentence in L92 in the revised MS.

5. Line 75. Please indicate the periodicity.

>> We changed “periodically” to “2-3 times in a year”. Please see L77 in the revised MS.

6. Lines 84-96. Did you use 12 apples as a total experimental set? Six apples per treatment? Please clarify.

>> Yes. We added “with six replications (5 insects/ apple fruit).” Please see L95 in the revised MS.

7. Lines 100-109. Were the apples the experimental unit? Or the larvae?

>> Apple fruits were the experimental unit and we revised the sentence. See L81 in the revised MS.

8. Five larvae per fruit gives 30 individual insects, some of them died so how many did the authors use for determining the demographic parameters?

>> We added the information in L110-113 in the revised MS.

Results

9. Figures can be deleted and the values presented on them can be included in the text.

>> We deleted Figure 2 as it is redundant data of Table 2.

D. Author’s corrections (Line numbers are from the first MS)

L114: We added “rate” after “survival” in L118 in the revised MS.

L115: We deleted “the percentages of larval and pupal mortality” in L119 in the revised MS.

L115: We changed “were” to “was” in L119 in the revised MS.

L134: We corrected “0.000” to “1.000” in L137 in the revised MS. It was type error.

L139-140: We changed “Age- and stage-specific survival rates of *G*. *molesta* larvae were highest on 20th day in both punctured and unpunctured apple fruits (Fig. 1)” to “The survival rates of *G*. *molesta* larvae reared on punctured and unpunctured apples were highest on 23rd and 21st day, respectively (Fig. 1)”. See L142-143 in the revised MS.

L150: We changed “Fig. 3” to “Fig. 2” in L151 in the revised MS.

L186: We deleted “because the larval and pupal mortality rates were lower in punctured fruits” in L187 in the revised MS.

L201: We changed “larval and pupal mortality” to “overall survival rate” in L202 in the revised MS.

L203: We added “(Table 1)” in L204 in the revised MS.

L204: We changed “larval” to “overall” in L205 in the revised MS.

L226: Changed “wprs” to “WPRS” in L229 in the revised MS.

L239: Changed “Sinica” to “Sin” in L242 in the revised MS.

L245 and L266: Changed “Manage” to “Manag” in L248 and L269 in the revised MS.

L312: Deleted “and”. See L315 in the revised MS.

L321: Changed “Chern” to “Chem” in L324 in the revised MS.
